# Protection against SARS-CoV-2 Omicron BA.1 variant challenge in macaques by prime-boost vaccination with Ad26.COV2.S and SpFN

**DOI:** 10.1126/sciadv.ade4433

**Published:** 2022-11-23

**Authors:** Jingyou Yu, Paul V. Thomas, Katherine McMahan, Catherine Jacob-Dolan, Jinyan Liu, Xuan He, David Hope, Elizabeth J. Martinez, Wei-Hung Chen, Michaela Sciacca, Nicole P. Hachmann, Michelle Lifton, Jessica Miller, Olivia C. Powers, Kevin Hall, Cindy Wu, Julia Barrett, Isabella Swafford, Jeffrey R. Currier, Jocelyn King, Courtney Corbitt, William C. Chang, Emily Golub, Phyllis A. Rees, Caroline E. Peterson, Agnes Hajduczki, Elizabeth Hussin, Camille Lange, Hua Gong, Gary R. Matyas, Mangala Rao, Dominic Paquin-Proulx, Gregory D. Gromowski, Mark G. Lewis, Hanne Andersen, Meredith Davis-Gardner, Mehul S. Suthar, Nelson L. Michael, Diane L. Bolton, M. Gordon Joyce, Kayvon Modjarrad, Dan H. Barouch

**Affiliations:** ^1^ Center for Virology and Vaccine Research, Beth Israel Deaconess Medical Center, Boston, MA 02215, USA.; ^2^ Emerging Infectious Diseases Branch, Walter Reed Army Institute of Research (WRAIR), Silver Spring, MD 20910, USA.; ^3^ Henry M. Jackson Foundation for the Advancement of Military Medicine, Bethesda, MD 20817, USA.; ^4^ Ragon Institute of MGH, MIT, and Harvard, Cambridge, MA 02215, USA.; ^5^ Harvard Medical School, Boston, MA 02215, USA.; ^6^ Viral Diseases Branch, Walter Reed Army Institute of Research, Silver Spring, MD 20910, USA.; ^7^ U.S. Military HIV Research Program, Walter Reed Army Institute of Research, Silver Spring, MD 20910, USA.; ^8^ Bioqual, Rockville, MD 20852, USA.; ^9^ Emory Vaccine Center, Emory University School of Medicine, Atlanta, GA 30329, USA.; ^10^ Center for Infectious Diseases Research, Walter Reed Army Institute of Research (WRAIR), Silver Spring, MD 20910, USA.

## Abstract

Emerging severe acute respiratory syndrome coronavirus 2 (SARS-CoV-2) variants and waning immunity call for next-generation vaccine strategies. Here, we assessed the immunogenicity and protective efficacy of two SARS-CoV-2 vaccines targeting the WA1/2020 spike protein, Ad26.COV2.S (Ad26) and Spike ferritin Nanoparticle (SpFN), in nonhuman primates, delivered as either a homologous (SpFN/SpFN and Ad26/Ad26) or heterologous (Ad26/SpFN) prime-boost regimen. The Ad26/SpFN regimen elicited the highest CD4 T cell and memory B cell responses, the SpFN/SpFN regimen generated the highest binding and neutralizing antibody responses, and the Ad26/Ad26 regimen generated the most robust CD8 T cell responses. Despite these differences, protective efficacy against SARS-CoV-2 Omicron BA.1 challenge was similar for all three regimens. After challenge, all vaccinated monkeys showed significantly reduced peak and day 4 viral loads in both bronchoalveolar lavage and nasal swabs as compared with sham animals. The efficacy conferred by these three immunologically distinct vaccine regimens suggests that both humoral and cellular immunity contribute to protection against SARS-CoV-2 Omicron challenge.

## Teaser

This paper reports protection against SARS-CoV-2 Omicron by Ad26.COV2.S and SpFN vaccines in macaques.

## INTRODUCTION

The rapid development and deployment of safe and effective coronavirus disease 2019 (COVID-19) vaccines moderated the extent of the COVID-19 pandemic (*1*). However, this scientific triumph has been challenged by the emergence of a succession of severe acute respiratory syndrome coronavirus 2 (SARS-CoV-2) variants of concern, many of which escape commonly elicited immune responses, blunting the efficacy of first-generation SARS-CoV-2 vaccines and host antiviral defenses and others that affect viral fitness, transmissibility, and pathogenesis (*2*, *3*). This has been reflected by variants such as Alpha, Delta, and successive Omicron subvariants. In each wave, new variants demonstrated greater transmissibility and/or stronger immune evasion capacity and thus higher frequency of breakthrough infections (*4*, *5*). Therefore, next-generation vaccine strategies that elicit immunity with higher potency, expanded breadth, and greater durability are needed.

Heterologous, or mix-and-match, prime-boost immunization, when given with different delivery approaches but with matched antigen, represents a strategy to increase vaccine immunogenicity (*6*). Historically, vaccines against several infectious pathogens including HIV-1, Ebola, and influenza have been extensively investigated by multiple delivery methods, including various combinations of DNA vaccines, viral vectored vaccines, subunit vaccines, and others (*7*–*9*). An HIV-1 vaccine study showed that heterologous DNA prime-protein boost induced qualitatively and quantitatively superior antigen-specific antibody responses than DNA or protein only regimen (*10*). For COVID-19 vaccines, “mix-and-match” regimens have been authorized by the Food and Drug Administration (*11*) and have been shown to induce robust humoral and cellular immune responses (*6*).

Ad26.COV2.S is an emergency use authorization–approved recombinant replication-incompetent viral vectored vaccine that expresses a prefusion stabilized SARS-CoV-2 spike protein from the prototype WA1/2020 strain (*12*, *13*). A single shot of Ad26.COV2.S immunization induced high CD8 T cell responses and long-lasting general immune responses in animal models and humans (*12*–*15*). Yet, lower antibody responses are observed with Ad26.COV2.S in the early postvaccination period when compared with other vaccine modalities (*16*, *17*). A spike ferritin nanoparticle (SpFN)–based subunit vaccine candidate currently in a phase 1 trial has been shown to elicit high levels of antibody responses in animal models (*18*–*21*). SpFN is composed of 24 copies of SARS-CoV-2 spike from the WA1/2020 strain with each monomer fused to *Helicobacter pylori* ferritin, which self-assembles to present eight spike trimers on a soluble nanoparticle. This geometric presentation of repetitive and appropriately spaced antigen on nanoscale particles enhances immunogenicity, possibly by increasing avidity to promote greater naïve B cell activation, memory B cell expansion, and long-lived plasma cell generation (*22*, *23*). Adjuvanted SpFN vaccine has been shown to induce both T helper 1 (T_H_1) and T_H_2 immune responses (*19*). Given the unique immunological features of each of these vaccines, we explored the immunogenicity and protective efficacy of vaccine regimens that included both Ad26 and SpFN against SARS-CoV-2 Omicron BA.1 challenge in macaques.

## RESULTS

### Ad26.COV2.S and SpFN immunogenicity

**Fig. 1.**
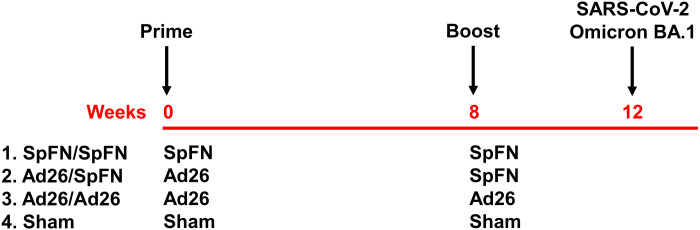
Study schema. Vaccine groups and timing of immunization and challenge are shown.

We immunized 24 cynomolgus macaques in four groups of six animals each (Fig. 1). All groups were primed at week 0 and boosted at week 8. Groups 1 and 3 received two homologous intramuscular immunizations with 5 μg of SpFN adjuvanted with Army Liposomal Formulation containing QS21 (ALFQ) or 5 × 10^10^ viral particles (vp) Ad26.COV2.S, respectively, reflecting the doses and formulations of each vaccine used in humans. Group 2 was primed with Ad26.COV2.S and boosted with SpFN. Group 4 received a sham vaccine. At week 12, all macaques were challenged with the SARS-CoV-2 Omicron variant BA.1.

**Fig. 2.**
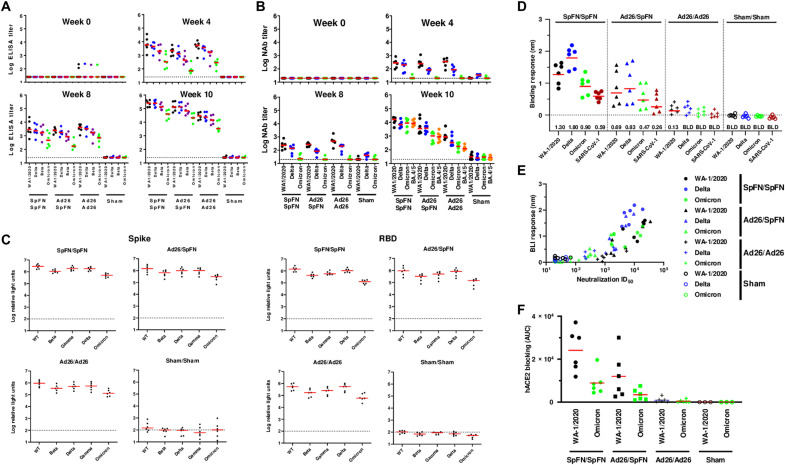
Humoral immune responses following vaccination. Antibody responses at weeks 0 (baseline), 4 (after prime), 8 (before boost), and 10 (after boost) following vaccination with SpFN/SpFN, Ad26/SpFN, Ad26/Ad26, or sham (*N* = 24; *N* = 6 per group). (**A**) RBD-specific endpoint binding antibody titers by ELISA. Responses were measured against the SARS-CoV-2 WA1/2020 (black), B.1.617.2 (Delta; blue), B.1.351 (Beta; purple), and B.1.1.529 (Omicron; green) variants. (**B**) Neutralizing antibody (NAb) titers (NT50) by a luciferase-based pseudovirus neutralization assay. (**C**) Spike- and RBD-specific binding antibody responses by MesoScale Discovery ECLA. Dotted lines represent limits of quantitation. Medians (red bars) are shown. Omicron-specific NAbs in the vaccinated groups were compared by two-sided Mann-Whitney tests. Only statistically significant differences, *P* < 0.05, are presented. (**D**) Biolayer mass interferometry (BLI) binding of sera to RBDs with colors as in (A) and (B) along with SARS-CoV-1 RBD (red) for SpFN/SpFN (closed circles), Ad26/SpFN (triangles), Ad26/Ad26 (crosses), and Sham/Sham (open circles). Geometric mean binding response is reported below with BLD indicating binding below the limit of detection of this assay. (**E**) BLI binding response plotted for each animal compared to their neutralization median inhibitory dose (ID_50_). (**F**) Human angiotensin converting enzyme 2 (ACE2)-RBD binding inhibition as measured by BLI and quantified by area under the curve (AUC) of reduction in BLI response for WA-1/2020 and Omicron RBDs. Colors indicate variant and shapes indicate groups as above.

We assessed humoral and cellular immune responses following Ad26.COV2.S and SpFN vaccination. Binding antibodies were assessed by receptor binding domain (RBD)–specific enzyme-linked immunosorbent assays (ELISAs) (*13*, *24*, *25*) as well as RBD- and spike-specific electrochemiluminescence assays (ECLAs) (*14*, *26*). Median RBD-specific ELISA titers at week 0 were below the limit of detection, except for two animals that showed a low background. At week 4 following SpFN prime vaccination, the median titers were 5824, 4403, 1985, and 323 against the WA1/2020, Delta, Beta, and Omicron BA.1 strains, respectively. In contrast, median titers at week 4 following Ad26 vaccination were 2150, 1297, 436, and 71 for Group 2 or 5291, 3625, 1667, and 286 for Group 3 against the WA1/2020, Delta, Beta, and Omicron BA.1 strains, respectively (Fig. 2A). Median ELISA titers at week 10 (2 weeks after boost) following SpFN/SpFN prime-boost vaccination were 252,863, 248,414, 132,305, and 39,808 against the WA1/2020, Delta, Beta, and Omicron BA.1 strains, respectively. The ELISA titers at week 10 following Ad26/SpFN vaccination were 131,794, 132,105, 67,875, and 20,718, while Ad26/Ad26 generated ELISA titers of 29,042, 23,891, 14,238, and 3410 against the WA1/2020, Delta, Beta, and Omicron BA.1 strains, respectively. The booster vaccination increased Omicron-specific ELISA titers by 122-fold (SpFN/SpFN), 291-fold (Ad26/SpFN), and 12-fold (Ad26/Ad26).

Neutralizing antibody (NAb) responses were determined by a luciferase-based pseudovirus NAb assay (*13*, *24*, *25*, *27*). Median inhibitory dose NAb titers at week 4 following SpFN prime vaccination were 283, 204, and 20 against the WA1/2020, Delta, and Omicron BA.1, respectively (Fig. 2B). At week 10, we included the currently circulating strain BA.4/5 variant for analysis, and the SpFN/SpFN regimen demonstrated median NAb titers of 16,322, 6872, 8780, and 8350.5 for WA1/2020, Delta, Omicron BA.1, and BA.4/5, respectively. The Ad26/SpFN regimen showed modestly lower median NAb titers of 3539, 3732, 1029, and 886 for WA1/2020, Delta, Omicron BA.1, and BA.4/5, respectively. The Ad26/Ad26 regimen elicited lower median NAb titers of 873, 333, 178, and 112 for WA1/2020, Delta, Omicron BA.1, and BA.4/5, respectively. Sham-immunized macaques demonstrated background levels of NAb titers. These data show that the boost immunization resulted in 1 to 2 log higher NAb titers against WA1/2020 and 1 to 3 log higher NAb titers against Omicron BA.1 (Fig. 2B). The SpFN/SpFN regimen induced the highest NAb titers.

We further analyzed antibody binding at peak immunity at week 10. Median RBD- and spike-specific ECLA titers were comparable for SpFN/SpFN and Ad26/SpFN, which trended higher than that of Ad26/Ad26 (Fig. 2C). Binding antibody titers were similar for full spike proteins from the matched variant WA1/2020 and the mismatched variants Beta, Delta, and Gamma, although lower binding was observed for Omicron BA.1. This pattern was accentuated for RBD-specific binding.

To better understand differences between these vaccine regimens, we used biolayer mass interferometry (BLI) to assay binding to sarbecovirus RBDs. This assay has a higher detection lower-limit and is therefore generally sensitive only to binding of higher affinity antibodies (*28*) (Fig. 2D). Again, SpFN/SpFN elicited the strongest binding across WA1/2020, Delta, and Omicron variants with only slightly less binding observed for Ad26/SpFN. Ad26/Ad26 was near the detection limit in this assay. We also assessed SARS-1 RBD binding (Fig. 2D). Ad26/SpFN elicited similar breadth to SpFN/SpFN with detectable binding to SARS-CoV-1. Comparing the BLI RBD-binding response of each variant to the neutralization titer, the Ad26/Ad26 group had a low overall slope, suggesting the ability to elicit a higher proportion of neutralizing to non-NAbs than SpFN/SpFN, which elicited higher neutralization and greater overall antibody binding (Fig. 2E). Serum from SpFN/SpFN- and Ad26/SpFN-vaccinated animals also blocked WA1/2020 RBD and, to a lesser extent, Omicron RBD from binding ACE2 in an in vitro BLI assay, while serum from Ad26/Ad26- and sham-vaccinated animals remained below the detectable limit for this assay (Fig. 2F).

Fc-mediated effector functions of antibodies elicited in each group were broadly similar at weeks 4 and 10 when measured against the prototype variant Wuhan-1 (fig. S1). However, at week 10, opsonization responses were higher for Ad26/SpFN compared to SpFN/SpFN, while antibody-dependent cellular cytotoxicity (ADCC) responses were higher for Ad26/Ad26 compared to SpFN/SpFN. Antibody-dependent cellular phagocytosis (ADCP) and antibody-dependent neutrophil phagocytosis (ADNP) responses at week 10 were higher for SpFN/SpFN and Ad26/SpFN compared to Ad26/Ad26. Antibody-dependent complement deposition (ADCD) responses were similar at week 10 for all three groups.

**Fig. 3.**
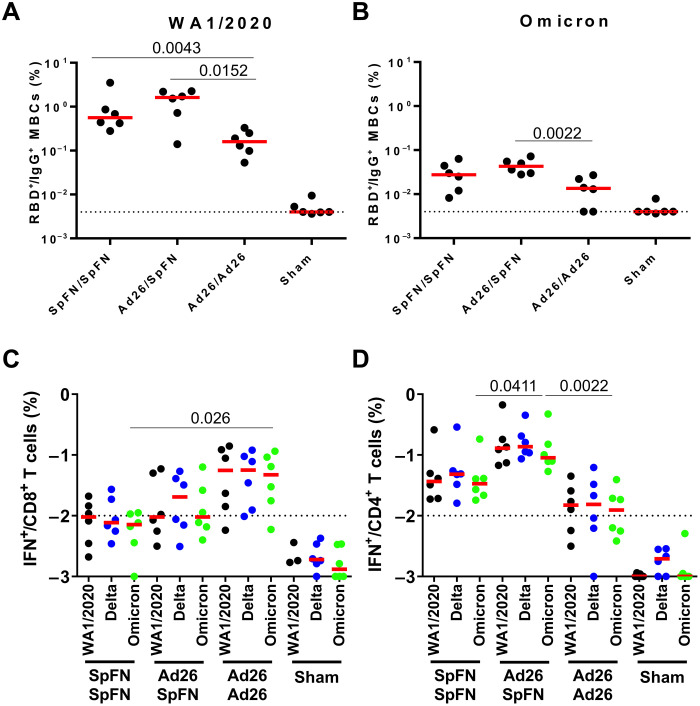
Cellular immune responses following vaccination. (**A** and **B**) RBD-specific B cell responses following vaccination. (A) Total WA1/2020 and (B) cross-reactive WA1/2020 and Omicron RBD-specific memory B cell responses in peripheral blood mononuclear cells (PBMCs) are shown by intracellular cytokine staining (ICS) assays. Dotted lines represent limits of quantitation. Medians (red bars) are shown. Vaccinated groups were compared by two-sided Mann-Whitney tests. Only statistically significant differences, *P* < 0.05, are presented. T cell responses at week 12 (after boost) and B cell responses at week 10 (after boost) following vaccination with SpFN/SpFN, Ad26/SpFN, Ad26/Ad26, or sham (*N* = 24; *N* = 6 per group). (**C** and **D**) Pooled peptide spike–specific IFN-γ (C) CD8^+^ T cell responses and (D) CD4^+^ T cell responses by ICS assays. Responses were measured against the SARS-CoV-2 WA1/2020 (black), B.1.617.2 (Delta; blue), and B.1.1.529 (Omicron; green) variants. Dotted lines represent limits of quantitation.

At week 12, we assessed memory immunoglobulin G (IgG)^+^ B cells in peripheral blood by multiparameter flow cytometry. Higher WA1/2020-specific RBD^+^ memory IgG^+^ B cell responses were observed in Ad26/SpFN-vaccinated animals compared with SpFN/SpFN and Ad26/Ad26 animals (Fig. 3A). A similar trend was observed in Omicron RBD^+^ memory IgG^+^ B cell responses (Fig. 3B).

Spike-specific CD8^+^ and CD4^+^ T cell responses were assessed by multiparameter flow cytometry in peripheral blood mononuclear cells (PBMCs) at week 12. Median Omicron spike-specific interferon-γ (IFN-γ)^+^ CD8^+^ T cell responses were 0.007, 0.009, 0.047, and 0.001% in the SpFN/SpFN, Ad26/SpFN, Ad26/Ad26, and sham groups, respectively (Fig. 3C). Median Omicron Spike-specific IFN-γ^+^ CD4^+^ T cell responses were 0.033, 0.090, 0.012, and 0.001% in the SpFN/SpFN, Ad26/SpFN, Ad26/Ad26, and sham groups, respectively (Fig. 3D). Omicron spike-specific IFN-γ CD8^+^ T cell responses were 6.6- and 5.0-fold higher in Ad26/Ad26 animals than in SpFN/SpFN and Ad26/SpFN animals, respectively. In contrast, the Omicron spike-specific IFN-γ^+^ CD4^+^ T cell responses were higher in Ad26/SpFN animals, about 2.7- and 7.3-fold higher than in the SpFN/SpFN and Ad26/Ad26 groups, respectively. As previously reported, T cell immune responses were highly cross-reactive across variants, with <2-fold differences for the WA1/2020, Delta, and Omicron BA.1 strains (Fig. 3, C and D) (*29*).

These data suggest that the heterologous Ad26/SpFN regimen–induced CD4 T cell and memory B cell responses were higher than the homologous SpFN/SpFN or Ad26/Ad26 regimens. However, the Ad26/SpFN regimen elicited lower CD8 T cell responses and lower NAb responses than the Ad26/Ad26 and SpFN/SpFN regimens, respectively.

### Protective efficacy against Omicron BA.1 challenge

**Fig. 4.**
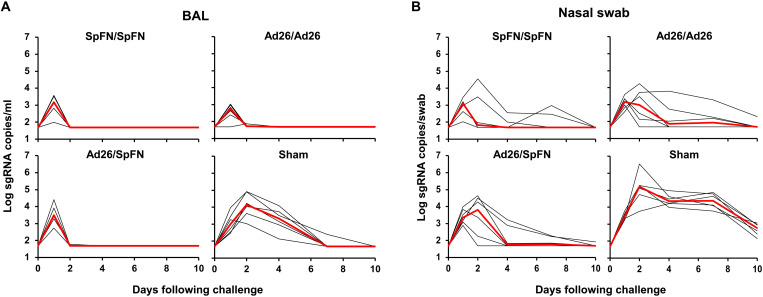
Viral loads following SARS-CoV-2 Omicron challenge. (**A**) Log sgRNA copies/ml in BAL following SARS-CoV-2 Omicron challenge. (**B**) Log sgRNA copies per swab in NS following SARS-CoV-2 Omicron challenge. Medians (red lines) are shown.

At week 12, all macaques were challenged with 1 × 10^6^ PFU (plaque-forming units) SARS-CoV-2 Omicron BA.1 by the intranasal and intratracheal routes. Protective efficacy was assessed following challenge in the upper and lower respiratory tract by monitoring subgenomic RNA (sgRNA) (*24*, *30*, *31*) in bronchoalveolar lavage (BAL) and nasal swabs (NSs) by reverse transcription polymerase chain reaction (RT-PCR). Sham controls showed median virus levels of 4.14 (range, 3.03 to 4.92) log sgRNA copies/ml in BAL on day 2, and these levels declined substantially by day 7 to median levels of less than 1.70 (range, 1.70 to 2.39) log sgRNA copies/ml (Fig. 4A). Vaccinated animals showed robust protection against Omicron, with nearly all vaccinated animals in BAL showing low and transient blips of sgRNA in BAL that largely resolved by day 2 following challenge (Fig. 4A).

In NS, sham controls showed median virus loads of 5.20 (range, 3.76 to 6.55) log sgRNA copies per swab on day 2, and these levels declined by day 7 to median levels of 4.38 (range, 3.77 to 4.87) log sgRNA copies per swab (Fig. 4B). All vaccinated animals were infected, and viral loads in NS were mostly resolved by day 4, with the exception of two to three animals in each group that showed persistent viral RNA in NS through days 7 to 10 (Fig. 4B).

**Fig. 5.**
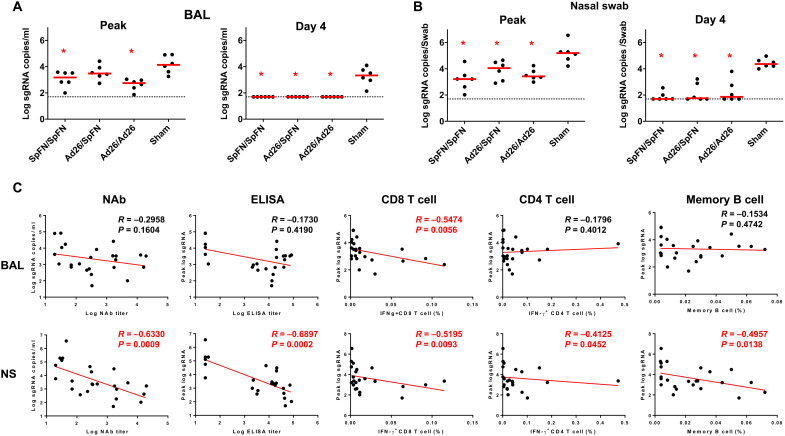
Comparison of peak and day 4 viral loads and correlation analysis. (**A**) Log sgRNA copies/ml in BAL at peak and on day 4 following SARS-CoV-2 Omicron challenge. (**B**) Log sgRNA copies per swab in NSs at peak and on day 4 following SARS-CoV-2 Omicron challenge. Dotted lines represent limits of quantitation. Medians (red bars) are shown. Vaccinated groups were compared with the sham controls by two-sided Mann-Whitney tests. *P < 0.05. (**C**) Correlations of week 10 NAb and ELISA titers and week 12 CD8^+^ and CD4^+^ T cell responses with peak sgRNA copies/ml in BAL and peak sgRNA copies per swab in NS are shown. Correlations were assessed by two-sided Spearman rank-correlation tests. *R* and *P* values and a regression line of best fit are shown.

Sham controls had higher median peak sgRNA levels of 4.14 (range, 3.26 to 4.92) log_10_ sgRNA copies/ml in BAL compared with vaccinated animals, which were reduced by 20.0-, 15.5-, and 32.2-fold in the SpFN/SpFN, Ad26/SpFN, and Ad26/Ad26 groups, respectively (*P* = 0.013, *P* = 0.179, and *P* = 0.002, respectively, two-tailed Mann-Whitney tests; Fig. 5A). At day 4 after challenge, sham controls showed median sgRNA levels of 3.35 (range, 2.14 to 4.10) log_10_ sgRNA copies/ml in BAL, while viral loads in BAL of vaccinated animals were reduced to undetectable levels <1.70 log_10_ sgRNA copies/ml in all groups (*P* = 0.002, *P* = 0.002, and *P* = 0.002, respectively, two-tailed Mann-Whitney tests; Fig. 5A). Similarly, sham controls had higher median peak sgRNA levels of 5.20 (range, 4.21 to 6.55) log_10_ sgRNA copies per swab in NS compared with vaccinated animals, which showed a median peak sgRNA of 3.42 (range, 3.21 to 4.05) log_10_ sgRNA copies per swab (Fig. 5B). At day 4 after challenge, sham animals demonstrated higher median sgRNA of 4.36 (range, 3.99 to 4.97) log_10_ sgRNA copies per swab in NS than vaccinated animals, which demonstrated undetectable or minimally detectable median log_10_ sgRNA copies per swab in all groups (Fig. 5B).

### Correlates of protection

The diversity of immune responses before challenge and viral loads following challenge allowed for a detailed immune correlates analysis. Peak log_10_ sgRNA in BAL or NS following challenge inversely correlated with Omicron NAb titers, ELISA titers, CD8^+^ T cell responses, CD4^+^ T cell responses, and B cell responses at week 10 or 12 (Fig. 5C). These data suggest that both humoral and cellular immunity contributed to virologic control following Omicron challenge.

## DISCUSSION

In this study, we demonstrated that SpFN and Ad26.COV2.S vaccines, administered in either homologous or heterologous regimens, led to rapid virologic control in the upper and lower respiratory tracts following high-dose, heterologous challenge with the SARS-CoV-2 Omicron BA.1 variant in macaques. Homologous regimens with SpFN/SpFN and Ad26/Ad26, as expected, elicited the strongest antibody responses and CD8 T cell responses, respectively. The heterologous Ad26/SpFN regimen induced the strongest CD4 T cell and memory B cell immune responses. This is particularly notable given the previously reported immunogenicity of the SpFN/SpFN vaccine (*20*).

It is well established that CD4 T cell responses serve not only as effectors that contribute to direct viral clearance but also as helpers that facilitate CD8^+^ T cell expansion and memory CD8^+^ T cell generation and orchestrate B cells to produce stronger and longer antibody responses (*32*). Long-lived antigen-specific memory B cells are important for generating anamnestic responses after antigen re-exposure and thus are critical in providing durable protection (*33*). Whether the particularly robust CD4^+^ T cell and B cell responses elicited by the heterologous Ad26/SpFN regimen leads to improved durability remains to be determined.

Immune correlates of protection analyses suggest that both humoral and cellular immune responses may contribute to protective efficacy against Omicron challenge in macaques. Omicron has been reported to replicate readily in the upper respiratory tract (*34*). It is possible that both antibodies and CD8 T cells control Omicron replication in the upper airway, whereas CD8 T cell responses likely contribute to viral clearance following lower respiratory tract infection.

Omicron infection occurred in both upper and lower respiratory tracts of nearly all animals, despite high antibody titers, suggesting that exceptionally high antibody titers may be required to achieve sterilizing immunity against this variant. All three vaccine regimens demonstrated similarly rapid virologic control, consistent with the model that multiple immunologic pathways may lead to viral clearance and protection against severe disease. In summary, our data show robust immunogenicity and protective efficacy by both homologous and heterologous regimens involving SpFN and Ad26.COV2.S and have important implications for understanding immune correlates of protection against SARS-CoV-2 variants. Future studies should evaluate the durability of these regimens as well as other prime-boost vaccine combinations.

## METHODS

### Animals and vaccines

Twenty-four outbred adult male and female cynomolgus macaques ages 4 to 12 years old were randomly allocated to four experimental groups (*N* = 6 per group; Fig. 1). All animals were singly housed at Bioqual Inc. (Rockville, MD). Groups of animals were primed with either 5 μg of SpFN (*19*) adjuvanted with ALFQ (*35*, *36*) or 5 × 10^10^ vp Ad26.COV2.S at week 0. At week 8, animals were boosted with either 5 μg of SpFN adjuvanted with ALFQ or 5 × 10^10^ vp Ad26.COV2.S. At week 12, all animals were challenged with 10^6^ PFU SARS-CoV-2 Omicron by the intranasal and intratracheal routes in a total volume of 2 ml. This challenge stock was generated in VeroE6-TMPRSS2 cells and had a titer of 2.3 × 10^9^ TCID_50_ (median tissue culture infectious dose)/ml and 2.5 × 10^7^ PFU/ml in VeroE6-TMPRSS2 cells and was fully sequenced (EPI_ISL_7171744; Mehul Suthar, Emory University). Following challenge, viral loads were assessed in BAL and NS samples by RT-PCR for E sgRNA. Animals were euthanized on day 9 or 10 following challenge. Immunologic and virologic assays were performed blinded. All animal studies were conducted in compliance with all relevant local, state, and federal regulations and were approved by the Bioqual Institutional Animal Care and Use Committee.

### Pseudovirus NAb assay

The SARS-CoV-2 pseudoviruses expressing a luciferase reporter gene were used to measure pseudovirus NAbs. In brief, the packaging construct psPAX2 (AIDS Resource and Reagent Program), luciferase reporter plasmid pLenti-CMV Puro-Luc (Addgene), and spike protein expressing pcDNA3.1-SARS-CoV-2 SΔCT were cotransfected into human embryonic kidney (HEK) 293T cells (American Type Culture Collection CRL_3216) with Lipofectamine 2000 (Thermo Fisher Scientific). Pseudoviruses of SARS-CoV-2 variants were generated by using the WA1/2020 strain (Wuhan/WIV04/2019, GISAID accession ID: EPI_ISL_402124), B.1.617.2 (Delta, GISAID accession ID: EPI_ISL_2020950), or B.1.1.529 (Omicron BA.1, GISAID ID: EPI_ISL_7358094.2). The supernatants containing the pseudotype viruses were collected 48 hours after transfection; pseudotype viruses were purified by filtration with a 0.45-μm filter. To determine the neutralization activity of human serum, HEK293T-hACE2 cells were seeded in 96-well tissue culture plates at a density of 1.75 × 10^4^ cells per well overnight. Threefold serial dilutions of heat-inactivated serum samples were prepared and mixed with 50 μl of pseudovirus. The mixture was incubated at 37°C for 1 hour before adding to HEK293T-hACE2 cells. After 48 hours, cells were lysed in Steady-Glo Luciferase Assay (Promega) according to the manufacturer’s instructions. SARS-CoV-2 neutralization titers were defined as the sample dilution at which a 50% reduction (NT_50_) in relative light units (RLUs) was observed relative to the average of the virus control wells.

### Enzyme-linked immunosorbent assay

SARS-CoV-2 spike RBD-specific binding antibodies in serum were assessed by ELISA. Ninety-six–well plates were coated with similarly produced SARS-CoV-2 WA1/2020, B.1.617.2 (Delta), B.1.351 (Beta), or B.1.1.529 (Omicron) RBD protein (1 μg/ml) in 1× Dulbecco phosphate-buffered saline (DPBS) and incubated at 4°C overnight. After incubation, plates were washed once with wash buffer (0.05% Tween 20 in 1× DPBS) and blocked with 350 μl of casein block solution per well for 2 to 3 hours at room temperature. Following incubation, block solution was discarded, and plates were blotted dry. Serial dilutions of heat-inactivated serum diluted in Casein block were added to wells, and plates were incubated for 1 hour at room temperature, before three more washes and a 1-hour incubation with a 1 μg/ml dilution of anti–human IgG horseradish peroxidase (Invitrogen, Thermo Fisher Scientific) at room temperature in the dark. Plates were washed three times, and 100 μl of SeraCare KPL TMB SureBlue Start solution was added to each well; plate development was halted by adding 100 μl of SeraCare KPL TMB Stop solution per well. The absorbance at 450 nm was recorded with a VersaMax microplate reader (Molecular Devices). For each sample, the ELISA endpoint titer was calculated using a four-parameter logistic curve fit to calculate the reciprocal serum dilution that yields an absorbance value of 0.2 at 450 nm. Interpolated endpoint titers were reported.

### Electrochemiluminescence assay

ECLA plates [MesoScale Discovery (MSD) SARS-CoV-2 IgG, panels 22 and 23] were designed and produced with up to 10 antigen spots in each well, including spike and RBD from multiple SARS-CoV-2 variants. The plates were blocked with 50 μl of blocker A (1% bovine serum albumin in distilled water) solution for at least 30 min at room temperature shaking at 700 rpm with a digital microplate shaker. During blocking, the serum was diluted to 1:5000 in Diluent 100. The calibrator curve was prepared by diluting the calibrator mixture from the MSD assay 1:9 in Diluent 100 and then preparing a seven-step fourfold dilution series plus a blank containing only Diluent 100. The plates were then washed three times with 150 μl of wash buffer (0.5% Tween in 1× PBS) and blotted dry, and 50 μl of the diluted samples and calibration curve were added in duplicate to the plates and set to shake at 700 rpm at room temperature for at least 2 hours. The plates were again washed three times, and 50 μl of SULFO-Tagged anti-human IgG detection antibody diluted to 1× in Diluent 100 was added to each well and incubated shaking at 700 rpm at room temperature for at least 1 hour. Plates were then washed three times, 150 μl of MSD GOLD Read Buffer B was added to each well, and the plates were read immediately after on a MESO QuickPlex SQ 120 machine. MSD titers for each sample were reported as RLUs that were calculated using the calibrator.

### ICS assay

CD4^+^ and CD8^+^ T cell responses were quantitated by pooled peptide–stimulated intracellular cytokine staining (ICS) assays. Peptide pools were 16–amino acid peptides overlapping by 11 amino acids spanning the SARS-CoV-2 WA1/2020, B.1.617.2 (Delta), or B.1.1.529 (Omicron; GISAID ID: EPI_ISL_7358094.2) spike proteins (21st Century Biochemicals). A total of 10^6^ PBMCs per well were resuspended in 100 μl of R10 medium supplemented with CD49d monoclonal antibody (1 μg/ml) and CD28 monoclonal antibody (1 μg/ml). Each sample was assessed with mock (100 μl of R10 plus 0.5% DMSO; background control), peptides (2 μg/ml), and/or phorbol myristate acetate (10 pg/ml) and ionomycin (1 μg/ml; Sigma-Aldrich) (100 μl; positive control) and incubated at 37°C for 1 hour. After incubation, 0.25 μl of GolgiStop and 0.25 μl of GolgiPlug in 50 μl of R10 were added to each well and incubated at 37°C for 8 hours and then held at 4°C overnight. The next day, the cells were washed twice with DPBS, stained with aqua live/dead dye for 10 min, and then stained with predetermined titers of monoclonal antibodies against CD279 (clone EH12.1, BB700), CD4 (clone L200, BV711), CD27 (clone M-T271, BUV563), CD8 (clone SK1, BUV805), and CD45RA [clone 5H9, allophycocyanin (APC) H7] for 30 min. Cells were then washed twice with 2% fetal bovine serum (FBS)/DPBS buffer and incubated for 15 min with 200 μl of BD CytoFix/CytoPerm Fixation/Permeabilization solution. Cells were washed twice with 1× Perm Wash buffer (BD Perm/Wash Buffer 10× in the CytoFix/CytoPerm Fixation/Permeabilization kit diluted with MilliQ water and passed through a 0.22-μm filter) and stained intracellularly with monoclonal antibodies against Ki67 (clone B56, BB515), interleukin-21 (IL-21) [clone 3A3-N2.1, phycoerythrin (PE)], CD69 [clone TP1.55.3, Electron Coupled Dye (ECD)], IL-10 (clone JES3-9D7, PE CY7), IL-13 (clone JES10-5A2, BV421), IL-4 (clone MP4-25D2, BV605), tumor necrosis factor–α (clone Mab11, BV650), IL-17 (clone N49-653, BV750), IFN-γ (clone B27, BUV395), IL-2 (clone MQ1-17H12, BUV737), IL-6 (clone MQ2-13A5, APC), and CD3 (clone SP34.2, Alexa Fluor 700) for 30 min. Cells were washed twice with 1× Perm Wash buffer and fixed with 250 μl of freshly prepared 1.5% formaldehyde. Fixed cells were transferred to 96-well round bottom plate and analyzed by the BD FACSymphony system. Data were analyzed using FlowJo v9.9.

### B cell staining

Fresh PBMCs were stained with Aqua live/dead dye (Invitrogen) for 20 min, washed with 2% FBS/DPBS buffer, and suspended in 2% FBS/DPBS buffer with Fc Block (BD Biosciences) for 10 min, followed by staining with monoclonal antibodies against CD45 (clone D058-1283, BUV805), CD3 (clone SP34.2, APC-Cy7), CD7 (clone M-T701, Alexa Fluor 700), CD123 (clone 6H6, Alexa Fluor 700), CD11c (clone 3.9, Alexa Fluor 700), CD20 (clone 2H7, PE-Cy5), IgA (goat polyclonal antibodies, APC), IgG (clone G18-145, BUV737), IgM (clone G20-127, BUV396), IgD (goat polyclonal antibodies, PE), CD80 (clone L307.4, BV786), CD95 (clone DX2, BV711), CD27 (clone M-T271, BUV563), CD21 (clone B-ly4, BV605), and CD14 (clone M5E2, BV570). Cells were also stained with SARS-CoV-2 antigens including biotinylated SARS-CoV-2 RBD proteins (Sino Biological) and SARS-CoV-2 RBD proteins (Sino Biological) labeled with fluorescein isothiocyanate (FITC), DyLight 405, or APC (DyLight 405 Conjugation Kit, FITC Conjugation Kit, and APC Conjugation Kit, Abcam), at 4°C for 30 min. After staining, cells were washed twice with 2% FBS/DPBS buffer, followed by incubation with BV650 streptavidin (BD Pharmingen) for 10 min, and then washed twice with 2% FBS/DPBS buffer. After staining, cells were washed and fixed by 2% paraformaldehyde. All data were acquired on a BD FACSymphony flow cytometer. Subsequent analyses were performed using FlowJo software (TreeStar, v10.8.1). Immunologic assays were performed blinded.

### Subgenomic RT-PCR assay

SARS-CoV-2 E gene sgRNA was assessed by RT-PCR using primers and probes as previously described (*27*). A standard was generated by first synthesizing a gene fragment of the subgenomic E gene. The gene fragment was subsequently cloned into a pcDNA3.1^+^ expression plasmid using restriction site cloning (Integrated DNA Technologies). The insert was in vitro transcribed to RNA using the AmpliCap-Max T7 High Yield Message Maker Kit (CellScript). Log dilutions of the standard were prepared for RT-PCR assays ranging from 1 × 10^10^ to 1 × 10^–1^ copies. Viral loads were quantified from BAL fluid and NSs. RNA extraction was performed on a QIAcube HT using the IndiSpin QIAcube HT Pathogen Kit according to the manufacturer’s specifications (Qiagen). The standard dilutions and extracted RNA samples were reverse-transcribed using the SuperScript VILO Master Mix (Invitrogen) following the cycling conditions described by the manufacturer. A TaqMan custom gene expression assay (Thermo Fisher Scientific) was designed using the sequences targeting the E gene sgRNA. The sequences for the custom assay were as follows: forward primer, sgLeadCoV2.Fwd: CGATCTCTTGTAGATCTGTTCTC, E_Sarbeco_R: ATATTGCAGCAGTACGCACACA, and E_Sarbeco_P1 (probe): VIC-ACACTAGCCATCCTTACTGCGCTTCG-MGBNFQ. Reactions were carried out in duplicate for samples and standards on the QuantStudio 6 and 7 Flex Real-Time PCR Systems (Applied Biosystems) with the following thermal cycling conditions: initial denaturation at 95°C for 20 s, and then 45 cycles of 95°C for 1 s and 60°C for 20 s. Standard curves were used to calculate sgRNA copies/ml or per swab. The quantitative assay sensitivity was determined as 50 copies/ml or per swab.

### Biolayer interferometry

His-tagged sarbecovirus spike protein RBD molecules were produced, and the biolayer interferometry assays were performed as previously described (*19*). In brief, FortéBio (Fremont, CA, USA) HIS1K biosensors were hydrated in PBS before use. Sarbecovirus RBD molecules (30 μg/ml diluted in PBS) were allowed to load on the probes for 120 s. After briefly dipping in assay buffer (15 s in PBS), the biosensors were dipped in non-human primate sera collected at weeks 0 and 10 (100-fold dilution) for 180 s, before dissociation in PBS for 60 s. Binding response (in nanometers) is reported for the 180-s time point. The average binding response from serum collected at week 0 was used as a baseline and subtracted from binding response at week 10. For ACE2 inhibition assays, sera were serially diluted starting from a 25-fold dilution to a 3200-fold dilution. Sarbecovirus RBD molecules were immobilized as above and incubated in serially diluted sera for 200 s, followed by baseline equilibration (30 s), and then incubation with ACE2-Fc (30 μg ml^−1^) for 120 s. Zero percent inhibition was determined by a no-serum control; however, at high serum concentrations, up to 25% ACE2 binding inhibition was observed for the sham group. Inhibition is reported as area under the curve over the 25% inhibition baseline for each dilution versus dilution factor. All assays were performed at 30°C with agitation set at 1000 rpm.

### Antibody-dependent cellular phagocytosis

ADCP was measured as previously described (*37*). Briefly, biotinylated SARS-CoV-2 spike proteins from D614G and BA.1 (both from Sino Biological) were incubated with yellow-green streptavidin-fluorescent beads (Molecular Probes) for 2 hours at 37°C. Ten microliters of a 100-fold dilution of beads–protein was incubated for 2 hours at 37°C with 100 μl of a 1000-fold diluted plasma samples before the addition of THP-1 cells (25,000 cells per well; MilliporeSigma, Burlington, MA, USA). After 19 h of incubation at 37°C, the cells were fixed with 2% formaldehyde solution (Tousimis, Rockville, MD, USA), and fluorescence was evaluated on an LSR II (BD Biosciences). The phagocytic score was calculated by multiplying the percentage of bead-positive cells by the geometric mean fluorescence intensity (MFI) of the bead-positive cells and dividing the product by 10^4^.

### Antibody-dependent neutrophil phagocytosis

Biotinylated SARS-CoV-2 spike proteins from D614G and BA.1 variants were incubated with yellow-green streptavidin-fluorescent beads (Molecular Probes) for 2 hours at 37°C. Ten microliters of a 100-fold dilution of beads–protein was incubated for 2 hours at 37°C with 100 μl of a 1000-fold diluted plasma samples before the addition of effector cells (50,000 cells per well). Fresh peripheral blood leukocytes from human were used as effector cells after red blood cell lysis with Ammonium-Chloride-Potassium lysing buffer (Thermo Fisher Scientific). After 1 hour of incubation at 37°C, the cells were washed, surface-stained, and fixed with 4% formaldehyde solution (Tousimis, Rockville, MD, USA), and fluorescence was evaluated on an LSR II (BD Biosciences). Antibodies used for flow cytometry were anti-human CD3 AF700 (clone UCHT1) and anti-human CD14 APC-Cy7 (clone MϕP9; both from BD Biosciences) and anti-human CD66b Pacific Blue (clone G10F5, BioLegend). The ADNP phagocytic score was calculated by multiplying the percentage of bead-positive neutrophils (side-scatter (SSC) high, CD3^−^CD14^−^CD66^+^) by the geometric MFI of the bead-positive cells and dividing the product by 10^4^.

### Antibody-dependent complement deposition

SARS-CoV-2 spike-expressing expi293F cells were generated by transfection with linearized plasmid (pcDNA3.1) encoding codon-optimized full-length SARS-CoV-2 Spike protein matching the amino acid sequence of the IL-CDC-IL1/2020 isolate (GenBank, ACC# MN988713) or B.1.1.529. Stable transfectants were single-cell–sorted and selected to obtain a high-level spike surface expressing clone. An ADCD assay was adapted from (*38*). Briefly, 293F-Spike–expressing cells were incubated with 10-fold diluted heat-inactivated (56°C for 30 min) plasma samples for 30 min at 37°C. Cells were washed twice and resuspended in RPMI with 10% FBS. Lyophilized guinea pig complement (CL4051, Cedarlane, Burlington, Canada) was reconstituted per the manufacturer’s instructions in 1 ml of cold water and centrifuged at 13,000 rpm for 5 min at 4°C. Cells were washed with PBS and resuspended in 200 μl of guinea pig complement, which was prepared at a 1:50 dilution in Gelatin Veronal Buffer with Ca^2+^ and Mg^2+^ (IBB-300x, Boston BioProducts, Ashland, MA). After incubation at 37°C for 20 min, cells were washed in PBS with 15 mM EDTA (Thermo Fisher Scientific) and stained with an anti–guinea pig complement C3 FITC (polyclonal, Thermo Fisher Scientific). Cells were fixed with 4% formaldehyde solution, and fluorescence was evaluated on an LSR II (BD Biosciences).

### Opsonization

293F-spike–expressing cells were incubated with 100-fold diluted plasma samples for 30 min at 37°C. Cells were washed twice and stained with anti-human IgG PE, anti-human IgM Alexa Fluor 647, and anti-human IgA FITC (all from SouthernBiotech). Cells were then fixed with 4% formaldehyde solution, and fluorescence was evaluated on an LSR II (BD Biosciences).

### CD16 reporter assay (ADCC)

SARS-CoV-2 spike-expressing CEM cells were generated by transfection with linearized plasmid (pcDNA3.1) encoding codon-optimized full-length SARS-CoV-2 spike protein matching the amino acid sequence of the IL-CDC-IL1/2020 isolate (GenBank, ACC# MN988713) and B.1.1.529. Spike-expressing CEM cells were plated at 100,000 per well in round bottom 96-well plates and incubated with 100 μl of diluted plasma (100-fold) for 30 min at 37°C. Cells were washed, and 200,000 Jurkat-Lucia NFAT-CD16 cells (Invivogen) were added to each well in 100 μl of Iscove’s Modified Dulbecco’s Medium (IMDM) with 10% FBS. The cells were then centrifuged for 1 min at low speed and co-cultured for 24 hours at 37°C. Quanti-Luc (50 μl) was added to 20 μl of coculture supernatant, and luminescence was measured immediately on a luminometer (2104 Multilabel reader, PerkinElmer).

### Statistical analysis

Descriptive statistics and logistic regression were performed using GraphPad Prism 8.4.3 (GraphPad Software, San Diego, CA). Immunologic data were generated in duplicate and were compared by two-sided Mann-Whitney tests. Correlations were assessed by two-sided Spearman rank correlation tests. *P* values less than 0.05 were considered significant.
