# Supplementary material for: Protection against SARS-CoV-2 Omicron BA.1 variant challenge in macaques by prime-boost vaccination with Ad26.COV2.S and SpFN
Source: Sci Adv. 2022 Nov 23;8(47):eade4433. doi: 10.1126/sciadv.ade4433 (PMC9683731; doi:10.1126/sciadv.ade4433)
Supplement: Supplementary file 1 — Fig. S1 [file sciadv.ade4433_sm.pdf]

Science Advances
AAAS

Supplementary Materials for

Protection against SARS-CoV-2 Omicron BA.1 variant challenge in macaques by prime-boost vaccination with Ad26.COV2.S and SpFN

Jingyou Yu *et al.*

Corresponding author: M. Gordon Joyce, gjoyce@eidresearch.org; Dan H. Barouch, dbarouch@bidmc.harvard.edu

Sci. Adv. **8**, eade4433 (2022)
DOI: 10.1126/sciadv.ade4433

This PDF file includes:

Fig. S1

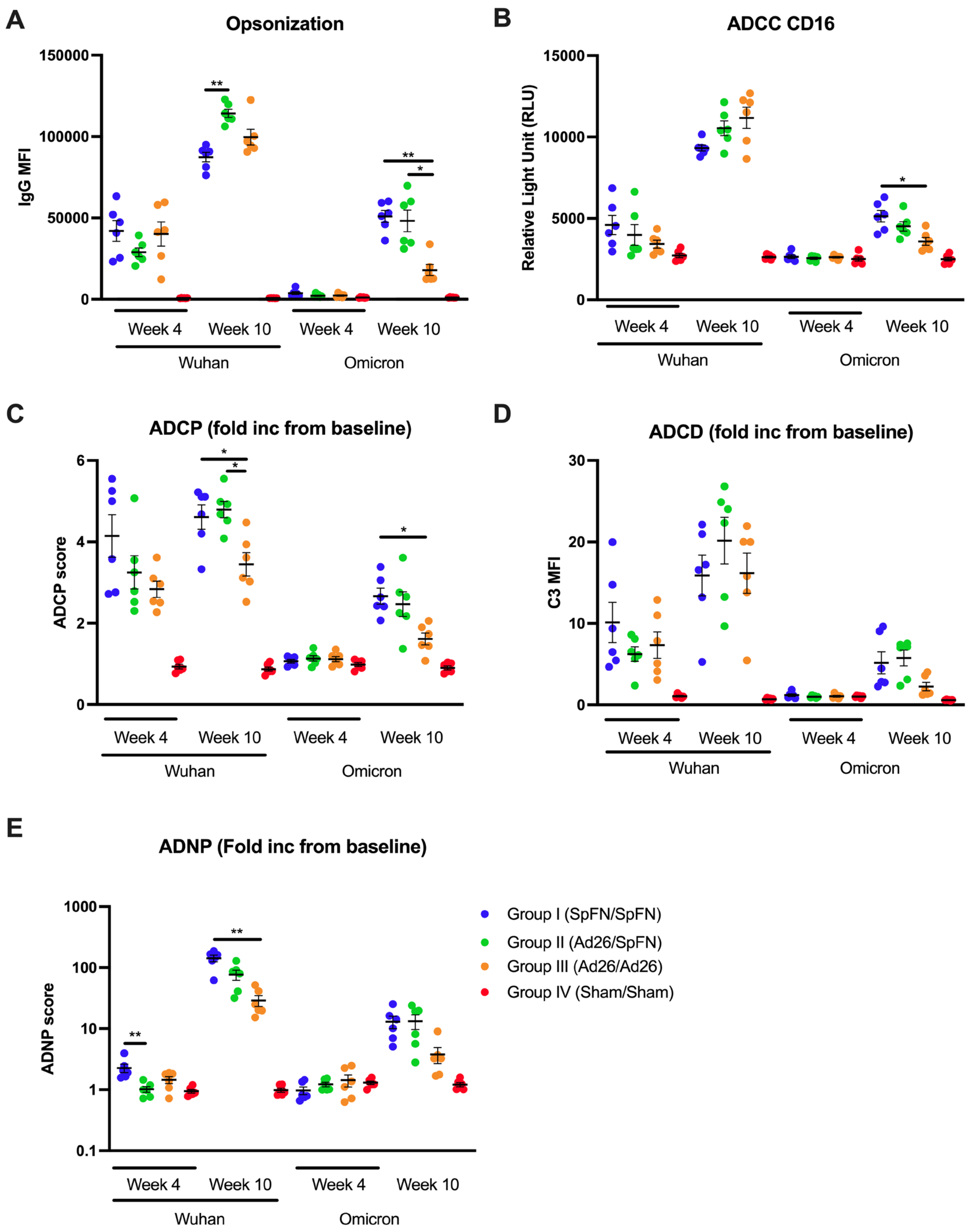
A
Opsonization
IgG MFI
150000
100000
50000
0
Week 4
Week 10
Week 4
Week 10
Wuhan
Omicron
B
ADCC CD16
Relative Light Unit (RLU)
15000
10000
5000
0
Week 4
Week 10
Week 4
Week 10
Wuhan
Omicron
C
ADCP (fold inc from baseline)
ADCP score
6
4
2
0
Week 4
Week 10
Week 4
Week 10
Wuhan
Omicron
D
ADCD (fold inc from baseline)
C3 MFI
30
20
10
0
Week 4
Week 10
Week 4
Week 10
Wuhan
Omicron
E
ADNP (Fold inc from baseline)
ADNP score
1000
100
10
1
0.1
Week 4
Week 10
Week 4
Week 10
Wuhan
Omicron
Group I (SpFN/SpFN)
Group II (Ad26/SpFN)
Group III (Ad26/Ad26)
Group IV (Sham/Sham)

Supplementary Figure Legends

Fig. S1. Fc-effector functions following vaccination. (A) Opsonization, **(B)**, antibody-dependent cellular toxicity (ADCC), **(C)** antibody-dependent cellular phagocytosis (ADCP), **(D)** antibody-dependent complement deposition (ADCD), and **(E)** antibody-dependent neutrophil phagocytosis (ADNP) measured at weeks 4 and 10 against the prototype Wuhan and Omicron BA.1 variants for SpFN/SpFN (blue), Ad26/SpFN (green), Ad26/Ad26 (orange), Sham/Sham (red). $*p < 0.05$, $**p < 0.01$, $***p < 0.001$, and $****p < 0.0001$.
